# The prognostic value of the NECPAL instrument, Palliative Prognostic Index, and PROFUND index in elderly residents of nursing homes with advanced chronic condition

**DOI:** 10.1186/s12877-023-04409-9

**Published:** 2023-11-03

**Authors:** Ana Alejandra Esteban-Burgos, César Hueso-Montoro, Emilio Mota-Romero, Rafael Montoya-Juarez, Xavier Gomez-Batiste, María Paz Garcia-Caro

**Affiliations:** 1 Departamento de Enfermería, Universidad de Jaén, Jaén, Spain; 2 Instituto Investigación Biosanitaria Granada (IBS), Granada, Spain; 3 Programa de Doctorado en Medicina Clínica y Salud Pública, Universidad de Granada, Granada, Spain; 4 Centro de Salud Salvador Caballero. Distrito Sanitario Granada-Metropolitano. Servicio Andaluz de Salud, Granada, Spain; 5 Departamento de Enfermería, Universidad de Granada, Granada, Spain; 6 Centro de Investigación Mente, Cerebro y Comportamiento (CIMCYC), Universidad de Granada, Granada, Spain; 7 Cátedra de Cuidados Paliativos, Universitat de Vic-Universitat Central de Catalunya (UVIC-UCC), Barcelona, Spain

**Keywords:** Palliative care, Prognosis, Nursing homes, Frail elderly, Aged, Palliative medicine, Geriatric assessment

## Abstract

**Background:**

It is essential to assess the need for palliative care and the life prognosis of elderly nursing home residents with an advanced chronic condition, and the NECPAL ICO-CCOMS©4.0 prognostic instrument may be adequate for both purposes. The objective of this study was to examine the predictive capacity of NECPAL, the Palliative Prognosis Index, and the PROFUND index in elderly residents with advanced chronic condition with and without dementia, comparing their results at different time points.

**Methods:**

This prospective observational study was undertaken in eight nursing homes, following the survival of 146 residents with advanced chronic condition (46.6% with dementia) at 3, 6, 12, and 24 months. The capacity of the three instruments to predict mortality was evaluated by calculating the area under the receiver operating characteristic curve (AUC), with 95% confidence interval, for the global population and separately for residents with and without dementia.

**Results:**

The mean age of residents was 84.63 years (± 8.989 yrs); 67.8% were female. The highest predictive capacity was found for PROFUND at 3 months (95%CI: 0.526–0.756; p = 0.016), for PROFUND and NECPAL at 12 months (non-significant; AUC > 0.5), and NECPAL at 24 months (close-to-significant (AUC = 0.624; 95% CI: 0.499–0.750; p = 0.053). The highest capacity at 12 months was obtained using PROFUND in residents with dementia (AUC = 0.698; 95%CI: 0.566–0.829; p = 0.003) and NECPAL in residents without dementia (non-significant; AUC = 0.649; 95%CI: 0.432–0.867; p = 0.178). Significant differences in AUC values were observed between PROFUND at 12 (p = 0.017) and 24 (p = 0.028) months.

**Conclusions:**

PROFUND offers the most accurate prediction of survival in elderly care home residents with advanced chronic condition overall and in those with dementia, especially over the short term, whereas NECPAL ICO-CCOMS©4.0 appears to be the most useful to predict the long-term survival of residents without dementia. These results support early evaluation of the need for palliative care in elderly care home residents with advanced chronic condition.

**Supplementary Information:**

The online version contains supplementary material available at 10.1186/s12877-023-04409-9.

## Introduction

The World Health Organization [1] has called for the implementation of an integrated care model for patients with chronic disease, combining specific therapy to prolong life with palliative care to relieve pain and suffering. Palliative care becomes more important with the progression of disease and is traditionally offered in its final stages, at the end of life. The integrated model is particularly designed for patients with long-term chronic diseases, including chronic obstructive pulmonary disease (COPD), heart or kidney failure, or neurodegenerative disease [1–3]. A key issue raised by this model is the timing of palliative care delivery [4, 5]. This challenge is addressed in the Catalan care model for patients with chronic diseases [6] by differentiating among complex chronicity, advanced chronic condition ( ACC), and terminal stage.

The life prognosis of patients is evidently crucial information; however, if it is the sole parameter considered, it might be erroneously concluded that palliative care is only necessary in the terminal stage of disease. Over the past few years, instruments have been developed to combine life prognosis estimation with the identification of palliative care needs [5, 7–9], yielding a more profound and realistic perspective [8–11]. Among these, NECPAL ICO-CCOMS© [12] represented a major advance by allowing identification of palliative needs of individuals defined as having advanced complex care disease (ACD). The current version (3.1) includes the question “Would you be surprised if the patient died over the next year?” for subjective assessment of the prognosis [11, 13, 14] as well as nine items on physical and psychosocial needs. Version 4.0 of this instrument was recently developed [8, 9, 15], preserving the “surprise” question and considering six of the same parameters to classify three prognostic stages for estimating patient survival. This version was tested in patients with different diseases in various clinical settings and offered a good prediction of survival at 24 months [9]. However, its performance has not been compared with that of other instruments used in patients with chronic disease, such as the Palliative Prognostic Index (PPI) [16] or PROFUND index [17–19]. It is also important to select instruments for prognostic estimation according to the disease and setting of patients [9, 16, 20–22]. This process is of particular interest in people with dementia, given the need to act early in accordance with the preferences of patients and to minimize invasive procedures at the end of life [23–32]. However, generic and nonspecific life prognosis evaluation tools have not demonstrated good predictive capacity in individuals with dementia [8, 9, 20].

Nursing home residents include many elderly individuals with dementia, which predisposes sufferers to institutionalization [33–36]. Reports on the prevalence of people with dementia in European nursing homes have ranged between 85.2% in Austria and 51.8% in Germany [37, 38]. In Spain, cognitive impairment was detected in 56.4% of residents of Andalusian nursing homes [10]. In general, nursing homes have only limited material, human, and training resources to meet the considerable demand for palliative care [39–41]; nevertheless, specific instruments may be required for residents with dementia, whose needs for palliative care may differ from those of residents without this condition [42].

The application of instruments with prognostic value that consider the presence of cognitive impairment and complex chronic disease can be crucial for planning the care of residents, and it is necessary to verify the most appropriate instrument for this purpose.

This article aimed to compare the capacity of three widely used prognostic instruments (NECPAL4.0, PPI, and PROFUND) to predict the survival of elderly nursing home residents with ACC over the short- and long-term, considering the presence/absence of diagnosed dementia.

## Methodology

### Design

This prospective observational study was performed in nursing homes, following the survival of study participants at 3, 6, 12, and 24 months.

### Participants and data gathering

The study population comprised residents of eight nursing homes in Granada and Jaen (Southern Spain) who participated in the Nursing Homes End of Life Program (NU-HELP) [10, 43]. Nurses with more than 6 months’ work experience and specifically trained in data gathering selected 20 residents at each center who had no end date for their stay in the home and met the following criteria of the Spanish Society of Palliative Care (SECPAL) for ACC [44]: the presence of advanced, progressive, and incurable disease with no reasonable possibility of therapeutic response; the presence of numerous conditions or intense, multiple, multifactorial, and changing symptoms; the cause of major emotional impact on patients, family members, and staff; and/or life expectancy of ≤ 6 months.

The designated nurses gathered data between March 2019 and March 2022, recording the survival of residents at 3, 6, 12, and 24 months post-enrolment. The research team were available by telephone to provide the nurses with any specific or complementary information required and made periodic visits to the centers to monitor the process. Informed consent was obtained from all participants in the study, being provided by representatives or appointed relatives of the residents with dementia. Out of the total of 160 residents selected for study, consent was not given by 11 residents or representatives, leaving a final sample of 149 residents.

### Variables and instruments

Table 1 reports information on the different instruments employed in this study. The main outcome variable was survival at 3, 6, 12, and 24 months post-enrolment.

**Table 1:** Instruments in the study

	NECPAL 3.1 [45]	NECPAL 4.0 [8, 9, 15]	PPI [16]	PROFUND [17, 18]
Objective	Evaluation of palliative needs	Palliative needs and prognosis evaluation	Prognosis evaluation	Prognosis evaluation
Group of patients	All	All	All	All
Setting	Primary Care, Nursing Home, Hospital Settings	Primary Care, Nursing Home, Hospital Settings	Primary Care, Nursing Home, Hospital Settings	Primary Care, Nursing Home, Hospital Settings
Surprise Question included	Yes	Yes	No	No
Indicators				
Palliative needs identified	Yes (Patient, relatives, professional)	Yes (Patient, relatives, professional)	No	No
Functional decline	Yes	Yes	Yes	Yes
Nutritional decline	Yes	Yes	Yes	No
Cognitive impairment	Yes	No	No	Yes
Severe dependency	Yes (Barthel/Karnofsky)	No	Yes (PPS)	Yes (Barthel)
Geriatric symptoms	Yes (All)	No	Yes (Delirium)	Yes (Delirium)
Other symptoms	Yes (Checklist ESAS)	No	Yes (Edemas, dyspnea)	No
Psychosocial problems	Yes (Emotional discomfort, social vulnerability)	No	No	Yes (Caregiver)
Multimorbidity	Yes	Yes	No	No
Use of resources	Yes	Yes (Hospital stays, treatment)	No	Yes (Hospitalization during previous year)
Severity indicators by disease	Yes	Yes	No	Yes
Other				Age and analytical parameters (Hemoglobin)
Results/Interpretations				
Palliative needs	Yes	Yes	No	No
Prognosis	No	•Stage I: median survival of 38 months •Stage II: median survival of 17.2 months •Stage III: median survival of 3.6 months.	% of deaths in patients by score: • 0–2 points: 40% deaths • 2–4: 42% deaths • 4–6: 47% deaths • 6-9.5: 53% deaths • 9.5: 68%: deaths	Risk of dying in 12 months: • 0–2 points (16% probability) • 3–7 points (22% probability) • > 7 points (34% probability)
Prognosis time period		38 months post-enrolment	180 days	1–4 years

### Data analysis

Quantitative variables were expressed as means with standard deviations and categorical variables as absolute frequencies and percentages. Variables were non-normally distributed according to the Kolmogorov-Smirnov test; therefore, non-parametric tests were applied, using the chi-square or Fisher test to verify independence among categorical variables and the Mann-Whitney U test to evaluate the association between independent samples (patients with and without dementia). The predictive capacity of the instruments for mortality was evaluated by calculating the area under the receiver operating characteristic curve (AUC) with 95% confidence interval. Analysis was performed for the global population and separately for residents with and without dementia. IBM SPSS v.25 © (IBM Corporation, Armonk, NY) was used for data analyses, considering p ≤ 0.05 to be significant.

### Ethical oversight

All participants or appointees (for patients with cognitive impairment) signed their informed consent. The study was approved by the Research Ethics Committee (AP-0105-2016), and data were treated in accordance with national data protection regulations [46, 47].

## Results

### Description of participants

**Fig. 1:**
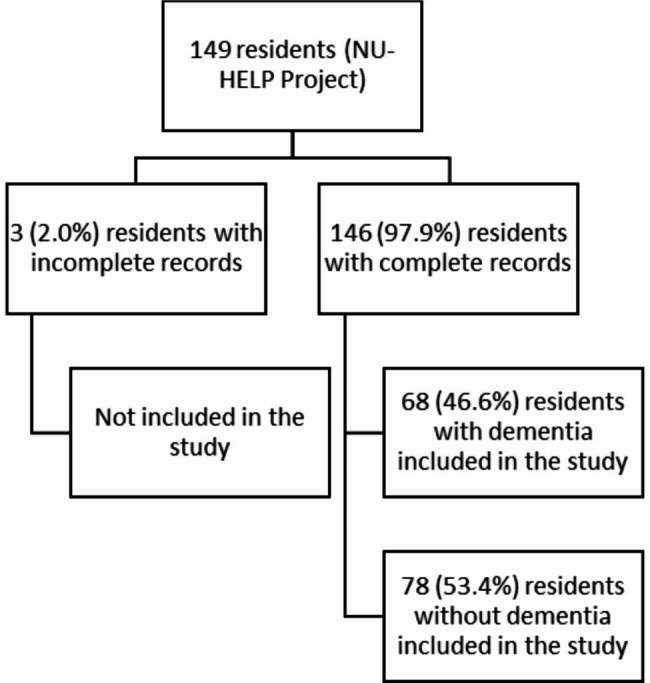
Flow diagram for the inclusion of cases in the analysis

Among the 149 residents selected from the NUHELP project [43], 146 had all records required for analyses (Fig. 1); the three residents (2%) with incomplete records were excluded from the study, leaving a final sample of 146 residents, including 68 (46.6%) with dementia.

The mean age of residents was 84.63 ± 8.989 years, and 99 were female (67.8%). After dementia (n = 68, 46.6%), the second most prevalent condition was chronic heart disease (CHD) (n = 57, 39%). Seventy-nine residents (54.1%) had ACD according to NECPAL ICO-CCOMS© 3.1. The mean PPI score was 2.53 (± 2.578), i.e., 42% of individuals with similar characteristics could be expected to die within six months (180 days). The mean PROFUND index score was 8.99 ± 3.996, indicating a mean expected survival of 9.3 months post-assessment.

Residents with and without dementia only significantly differed in the percentage with cancer (p < 0.001) COPD (p < 0.001), or CHD (p < 0.001), with CHD being the most prevalent comorbidity in both groups. They did not significantly differ in prognostic instrument scores or percentage with ACD by NECPAL (Table 2). Table 3 shows the survival stages of residents with ACD (n = 79) according to NECPAL-ICO-CCOMS©4.0.

**Table 2:** Description of the study population (N = 146)

Variables	Total Sample (n = 146) (m (sd) / md (P25–P75 / n(%))	Patients With Dementia (n = 68) (m (sd) / md (P25–P75 / n(%))	Patients Without Dementia (n = 78) (m (sd) / md (P25–P75 / n(%))	p
Age (yrs)	84.63 (± 8.989) / 87 (81.75-91)	84.68 (± 9.318) / 86 (83-91.75)	84.59 (± 8.753) / 88 (80–91)	0.381^a^
Female	99 (67.8)	48 (70.6)	51 (65.4)	0.502^b^
Comorbidities				
Cancer	24 (16.4)	3 (4.4)	21 (26.9)	0.000^b^
CPD	32 (21.9)	3 (4.4)	29 (37.2)	0.000^b^
CHD	57 (39)	11 (16.2)	46 (59)	0.000^b^
CLD	1 (0.7)	0 (0)	1 (1.3)	0.349^b^
CRD	18 (12.3)	7 (10.3)	11 (14.1)	0.485^b^
Dementia	68 (46.6)			
Clinical and Prognostic Assessment				
ACD (SQ+, NECPAL +)	79 (54.1)	29 (42.6)	38 (48.7)	0.463^b^
Palliative Prognostic Index	2.53 (± 2.578) / 2.5 (0–4)	2.88 (± 2.710) / 2.5 (0–4)	2.22 (± 2.434) / 1 (0-3.5)	0.959^a^
PROFUND	8.99 (± 3.996) / 9 (6–12)	9.21 (± 3.093) / 9 (8–12)	8.81 (± 4.654) / 9 (5–11)	0.399^a^

CPD, Chronic Pulmonary Disease; CHD, Chronic Heart Disease; CND, Chronic Neurological Disease; CLD, Chronic Liver Disease; CRD, Chronic Renal Disease; ACD, Advanced Chronic Disease; SQ+, positive response to Surprise Question; m, media; sd, standard deviation; ^a^Mann-Whitney; ^b^Chi-square

**Table 3:** NECPAL ICO-CCOMS(C)4.0 Survival stages of residents with ACD with and without dementia

Advanced Chronic Disease (ACD = SQ+, NECPAL+) (n = 79)	No + needs	3 (3.8)	*P* ^a^
	Est I	37 (46.8)	
	Est II	37 (46.8)	
	Est III	2 (2.5)	
ACD with dementia (n = 39)	No + needs	2(5.1)	0.459
	Est I	17 (43.6)	
	Est II	18 (46.2)	
	Est III	2 (5.1)	
ACD without dementia (n = 40)	No + needs	1 (2.5)	
	Est I	20 (50)	
	Est II	19 (47.5)	
	Est III	0 (0)	

^a^Chi-square test, SQ + = positive response to surprise question

### Comparison of mortality prediction between residents with and without Dementia

Fifty-six residents (38.4%) died during the follow up. The mean survival of deceased residents was 373.5 ± 222.328 days, with no significant differences between those with and without dementia. Thirty of these deaths (20.5% of total sample) occurred within 12 months. At the end of the follow-up, 45.6% of residents with dementia and 32.1% of those without dementia had died, although this difference was only statistically significant at three months (p = 0.004), when no deaths were recorded among residents without dementia (Table 4).

**Table 4:** Survival and prognostic index scores for all deceased residents and for those with and without dementia

		Deceased (n = 56)	With dementia (n = 31)	Without dementia (n = 25)	*p*
Survival in days m (sd) / md (P25–P75)		373.5 (± 222.328) / 320.5 (240.5-572.75)	358.7 (± 242.304) / 321 (179–558)	391.9 (± 198.119) / 315 (258-578.5)	0.604*
Deceased residents n = 56(%)	< 3 months	7 (12.5)	7 (22.6)	0 (0)	0.004**
	< 6 months	12 (21.4)	8 (25.8)	4 (16)	0.145**
	< 12 months	30 (53.6)	17 (54.8)	13 (52)	0.214**
	< 24 months	56 (100)	31 (100)	25 (100)	0.093**
ACD NECPAL-ICO-CCOMS©4.0 Deceased n = 30 (53.6%)	Stage I	11 (19.6)	7 (22.6)	4 (16)	0.734**
	Stage II	18 (32.1)	11 (35.5)	7 (28)	
	Stage III	1 (1.8)	1 (3.2)	0 (0)	
PPI score m (sd) / md (P25–P75)		2.55 (± 2.621) / 2.5 (0-3.5)	3 (± 2.652) / 2.5 (0-3.5)	2 (± 2.525) / 1 (0-3.5)	0.140*
PROFUND score m (sd) / md (P25–P75)		9.16 (± 4.080) / 9 (6.25-12)	10.06 (± 2.828) / 9 (9–12)	8.04 (± 5.078) / 9 (4-11.5)	0.051*

*Mann-Whitney; **Chi-Square

The majority of residents with ACC who had died were in survival stage II by NECPAL4.0 at three months (n = 18, 32.1%), while their mean PPI score was 2.55 (± 2.621) and mean PROFUND score 9.16 (± 4.080). Results of these instruments did not differ between deceased residents with and without dementia except for a close-to significant difference (p = 0.051) in PROFUND score (Table 4).

### Mortality prediction capacity of tools. ROC curves

**Fig. 2:**
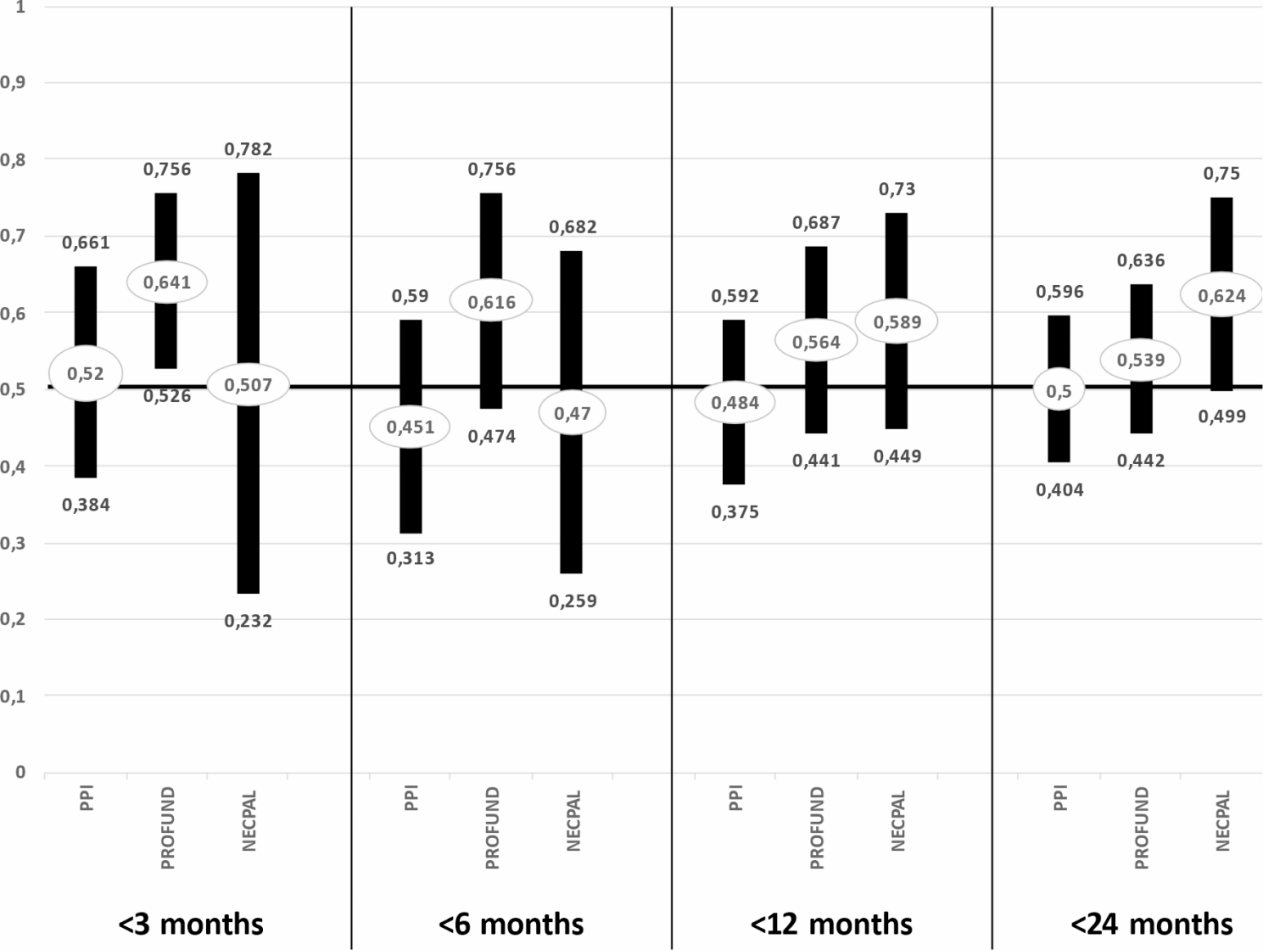
Area under the ROC curve obtained for NECPAL 4.0, PPI, and PROFUND *AUC; **95%CI; ◊*p* ≤ 0.05.

For the global sample, all three instruments had an AUC > 0.5 at three months. The best result was obtained with PROFUND at three months (95%CI: 0.526–0.756; *p* = 0.016) and again, although without statistical significance, at six months, (*p* = 0.106), while both PROFUND and NECPAL4.0 had an AUC > 0.5 at 12 months but without statistical significance (*p* = 0.310 and *p* = 0.212, respectively). All three instruments had an AUC > 0.5 at 24 months, when the best result was obtained using NECPAL, with a close-to-significant result (AUC = 0.624; 95%CI: 0.499–0.750; *p* = 0.053) (Fig. 2).

**Fig. 3:**
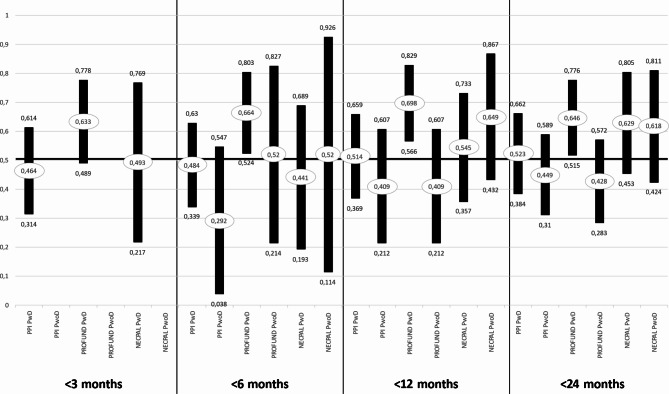
Area under the ROC curve of prognostic tools PPI, PROFUND, and NECPAL 4.0 inpatients with and without dementia *AUC; **95%CI; ◊*p* ≤ 0.05.

Among the residents with dementia, the highest AUC value was obtained with the PROFUND index at all time points, reaching statistical significance at 12 months (AUC = 0.698; 95%CI: 0.566–0.829; *p* = 0.003). Among those without dementia, the highest value was observed with NECPAL4.0 at 12 months (AUC = 0.649; 95%CI: 0.432–0.867; *p* = 0.178), although statistical significance was not reached. Residents with and without dementia significantly differed in AUC values for PROFUND at 12 (*p* = 0.017) and 24 (*p* = 0.028) months (Fig. 3). Data on the ROC curves of instruments for accumulated time periods are included as supplementary material. ROC curves of instruments at the different measurement time points for all patients who died and for those with and without dementia are given as supplementary material.

## Discussion

This study compared the predictive capacity of the new version 4.0 of the NECPAL instrument [8, 9, 15], PROFUND index [17, 18], and PPI [16, 48] at four time points (3, 6, 12, and 24 months) in a sample of elderly nursing home residents with ACC with and without dementia. Higher predictive capacities were found for NECPAL4.0 and PROFUND according to the presence/absence of dementia and measurement time point.

The age and sex profiles of the residents were similar to those in previous studies of this type in the nursing home setting [37, 49–55]. The percentage of residents classified with ACD by NECPAL3.1 was somewhat lower than described by Martínez-Muñoz [56] but higher than reported by da Costa et al. [57]. The percentage of residents with positive responses to the “surprise” question was higher than in previous studies that included this item in the study of other instruments [54, 55].

Similar proportions of residents with ACC were in NECPAL survival stages I and II and only a small proportion in stage III, implying a lower median survival (3.6 months). A higher proportion of patients were in stage III in the investigation by Calsina-Berna et al. [58], which included a larger percentage of patients receiving palliative care; however, few studies have been published on this issue, which warrants further research.

PROFUND results [7, 18] were in agreement with the findings by Da Costa et al. [57] and Moretti et al. [19] of a high or very high risk of mortality (≥ 7 points) in most residents. With regard to PPI findings, a mean of 4.5 points was obtained by Nieto-Martín et al. [16], indicating a worse survival than predicted in the present series.

There was a lower percentage of deaths in the present study than in some other studies of residents with dementia [23, 59, 60], although these only reported the mortality for periods < 12 months. Studies in nursing homes with a 24-month follow-up by Turrillas et al. [9] and Martínez-Muñoz et al. [56] observed a higher mortality rate (43% and 52.74%, respectively) at 24 months in comparison to the present series (38.4% ). Most residents who died within two years had dementia. Bernabeu-Wittel et al. [17] described the presence of dementia as a predictor of mortality and included it in the PROFUND index. In general, the instruments under study described a worse prognosis for the residents with *versus* without dementia, although dementia often coexists with other diseases that might affect the prognosis.

In the global sample, the highest AUC was obtained at 3 and 6 months using the PROFUND index [17, 18], with statistical significance, while the highest AUC at 24 months was obtained with NECPAL4.0, with a close-to-significant result, supporting data in the validation study for this instrument [9].

PROFUND obtained higher AUC values in the patients with *versus* without dementia, reaching statistical significance at 12 and 24 months. This might be attributable to the inclusion of a specific analytical parameter (hemoglobin < 10 g/dL) known to predict a worse prognosis and improve the prognostic accuracy in patients with different diseases, including dementia. NECPAL4.0 showed higher AUC values for the residents without dementia, in line with the finding by the original validation study of a worse predictive capacity in patients with *versus* without dementia [9]. The lowest AUC values were observed with PPI [16] at all follow-up times, and no statistically significant results were observed.

### Strengths and limitations

A larger sample of residents would have increased the statistical power, thereby improving the detection of statistical significance in trends identified in the present study. A further limitation was the lack of data on comorbidities in the groups with and without dementia, which should be provided separately in future studies, given that dementia is frequently accompanied by other diseases that can affect the prognosis. Only patients diagnosed with dementia were considered for this study. It should be considered for results interpretation that residents who have undiagnosed dementia or have some cognitive impairment have been excluded in the analysis. It should also be taken into account that part of the survival follow-up coincided with the COVID19 pandemic, although the nursing homes reported that none of the residents in the study died from this cause.

## Conclusions

According to these findings, PROFUND is the best instrument to predict survival in nursing home residents with ACC in general and in those with dementia, especially over the short term, whereas NECPAL4.0 offers the best performance in residents without dementia and over the long term (≥ 24 months). Hence, these instruments complement each other in terms of type of resident and time scale. NECPAL4.0 not only covers a longer time period than PROFUND but also offers information on the palliative needs of residents, helping nursing homes to prioritize resources for their adequate care.

A nursing home policy to estimate the prognosis of elderly people with ACC facilitates the early implementation of palliative care and supports multidimensional evaluation, advanced care planning, and resource management.

Combined evaluation of the care needs and the prognosis of residents provides a global view, whereas consideration of the prognosis alone may lead to the erroneous conclusion that palliative care is only necessary during the final stages of disease.

The detection of palliative care needs and application of a prognostic instrument with good predictive capacity in residents with dementia can improve their palliative care, reducing differences with dementia-free residents with ACC.

### Electronic supplementary material

Below is the link to the electronic supplementary material.

Supplementary Material 1

## Data Availability

The datasets analyzed during the current study are available from the corresponding author on reasonable request.

## Abbreviations

AUC: Area under the receiver operating characteristic curve
