# Supplementary material for: The prognostic value of the NECPAL instrument, Palliative Prognostic Index, and PROFUND index in elderly residents of nursing homes with advanced chronic condition
Source: BMC Geriatr. 2023 Nov 3;23:715. doi: 10.1186/s12877-023-04409-9 (PMC10623722; doi:10.1186/s12877-023-04409-9)
Supplement: Supplementary file 1 — Supplementary Material 1 [file 12877_2023_4409_MOESM1_ESM.docx]

Supplementary Material: ROC curves of instruments for accumulated time periods

Figure S1: ROC curve for NECPAL ICO-CCOMS©4.0, PPI and PROFUND in the whole sample up to 3 months


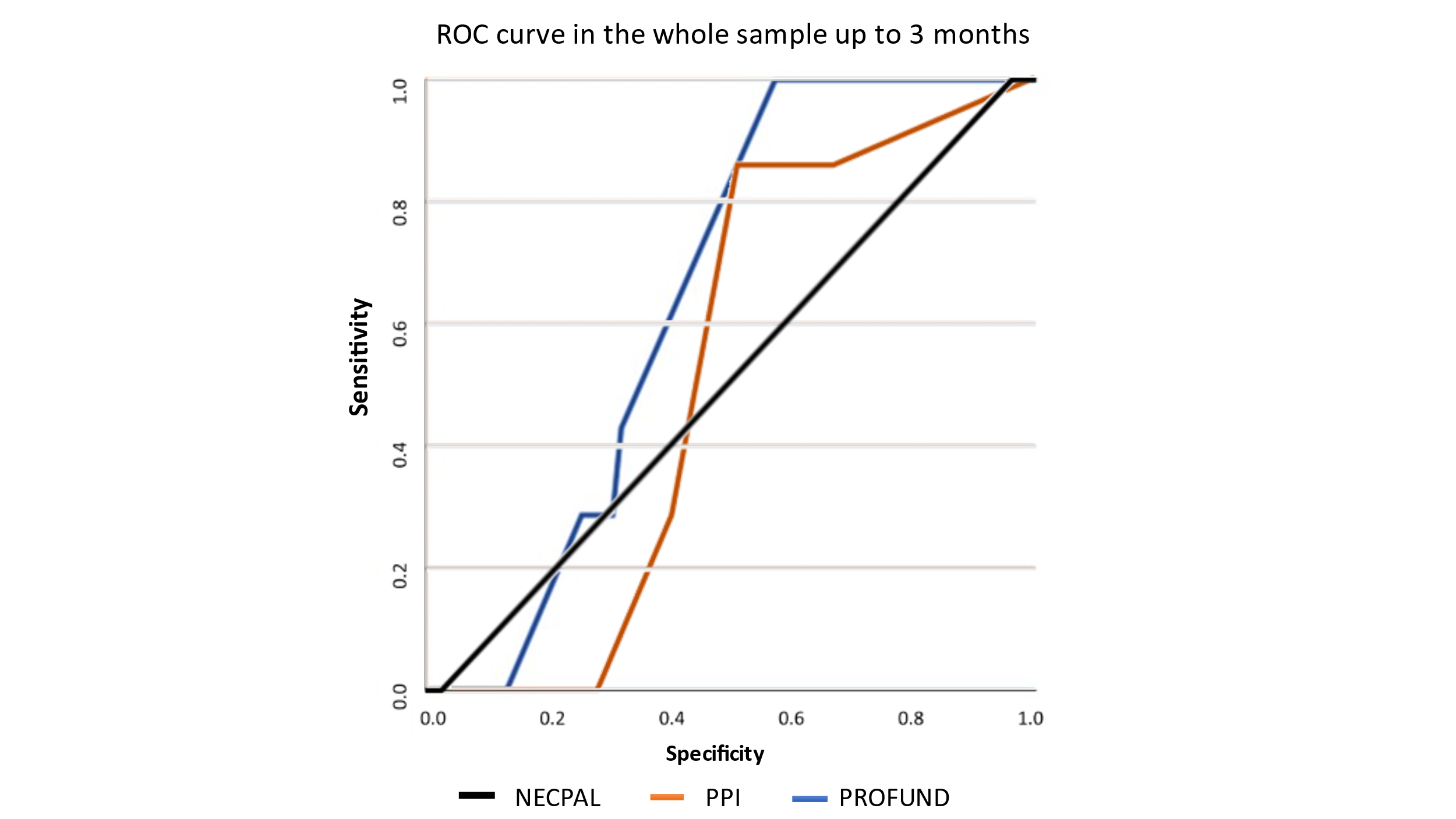


**Specificity**

Figure S2: ROC curve for NECPAL ICO-CCOMS©4.0, PPI and PROFUND in the whole sample up to 6 months.


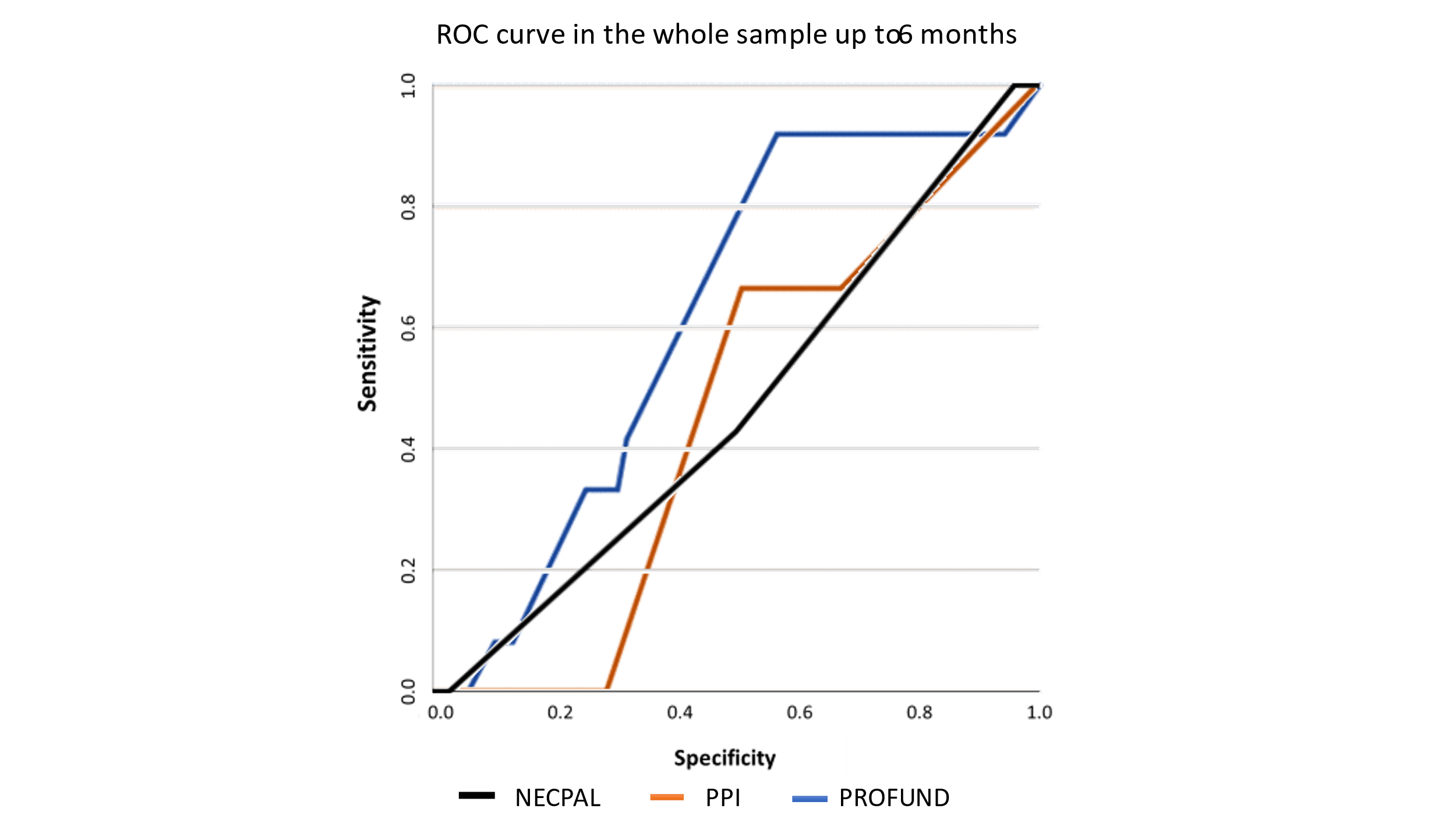


Figure S3: ROC curve for NECPAL ICO-CCOMS©4.0, PPI and PROFUND in the whole sample up to 12 months.


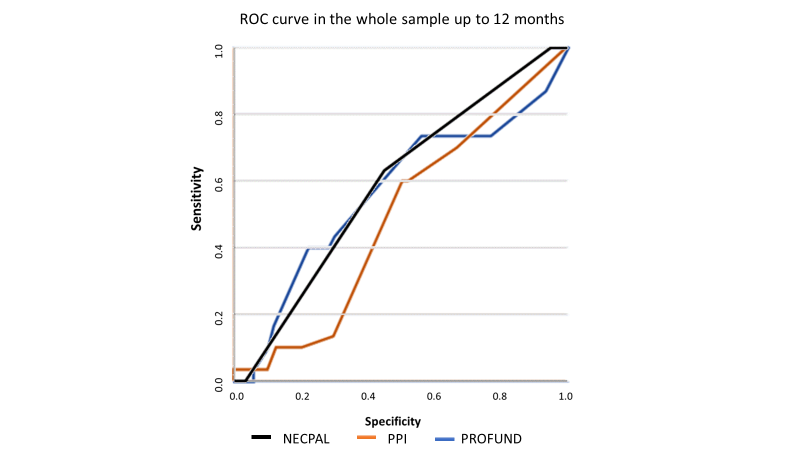


Figure S4: ROC curve for NECPAL ICO-CCOMS©4.0, PPI and PROFUND in the whole sample up to 24 months


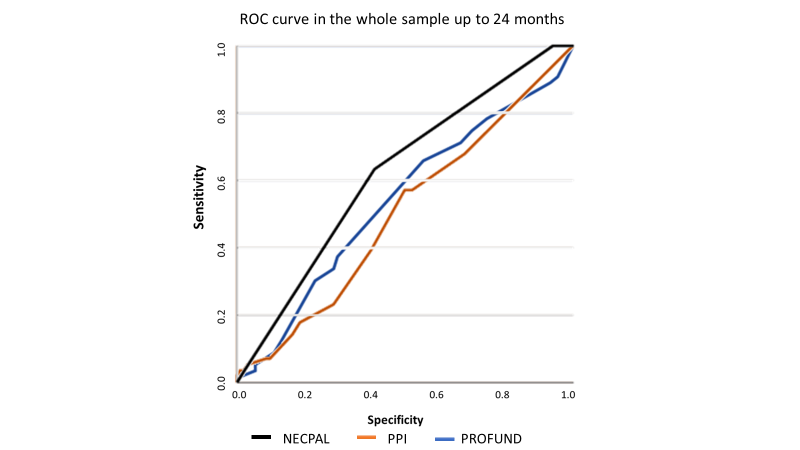


Figure S5: ROC curve for NECPAL ICO-CCOMS©4.0, PPI and PROFUND up to 3 months (Only residents with dementia).


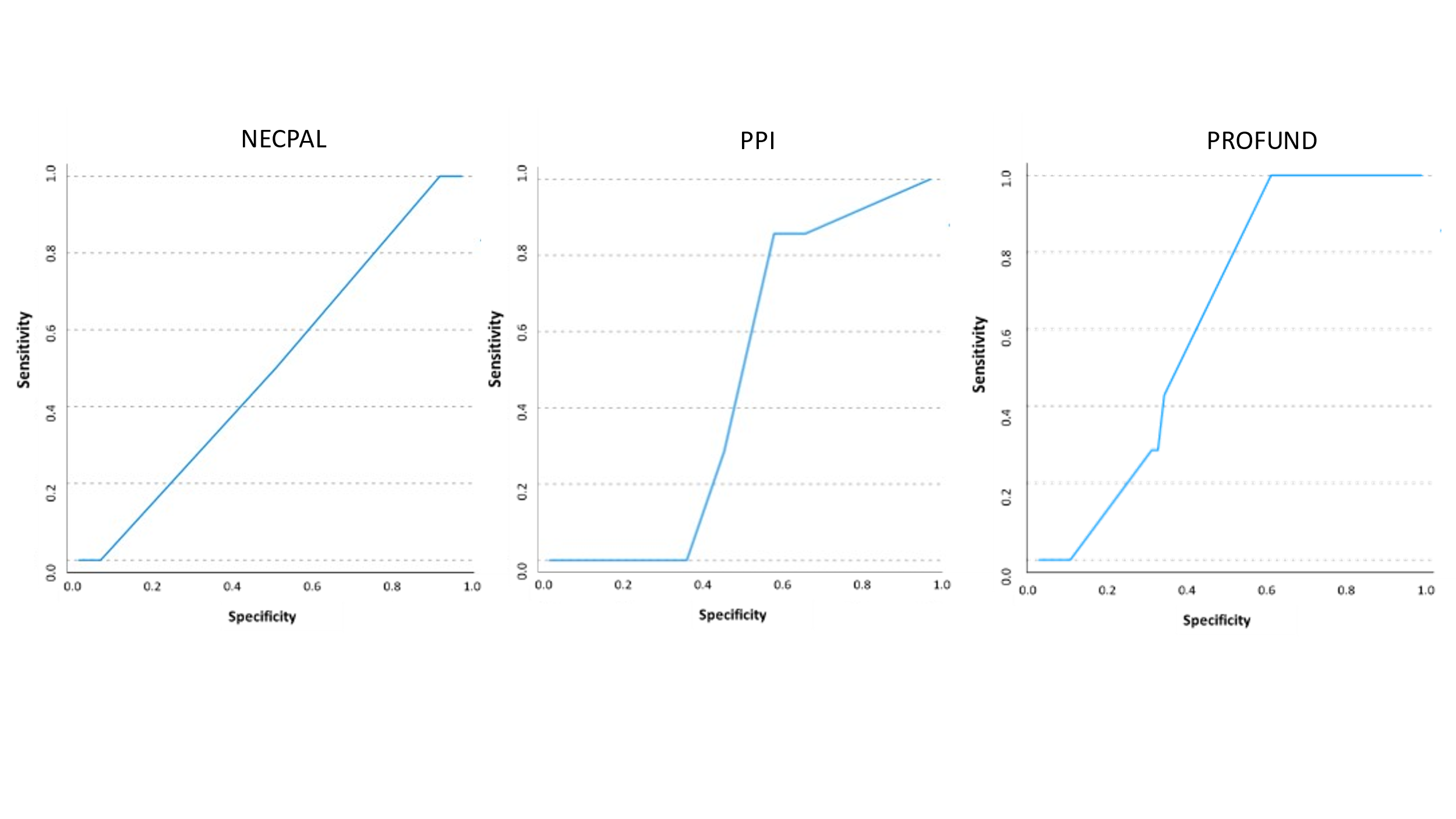


Figure S6: ROC curves for NECPAL ICO-CCOMS©4.0, PPI and PROFUND up to 6 months among residents with and without dementia.


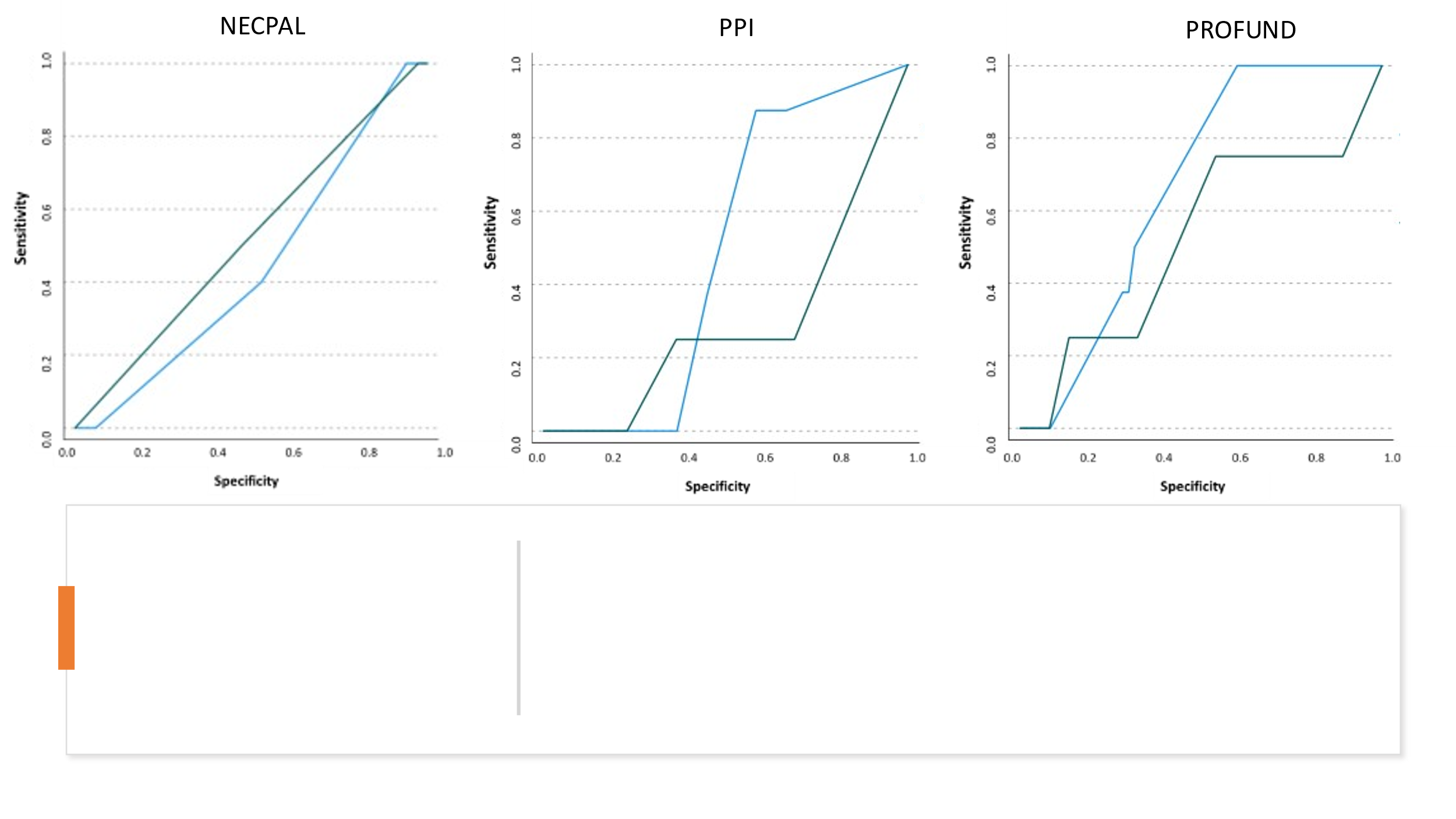


Green = Without dementia; Blue = With dementia

Figure S7: ROC curves for NECPAL ICO-CCOMS©4.0, PPI and PROFUND up to 12 months among residents with and without dementia.


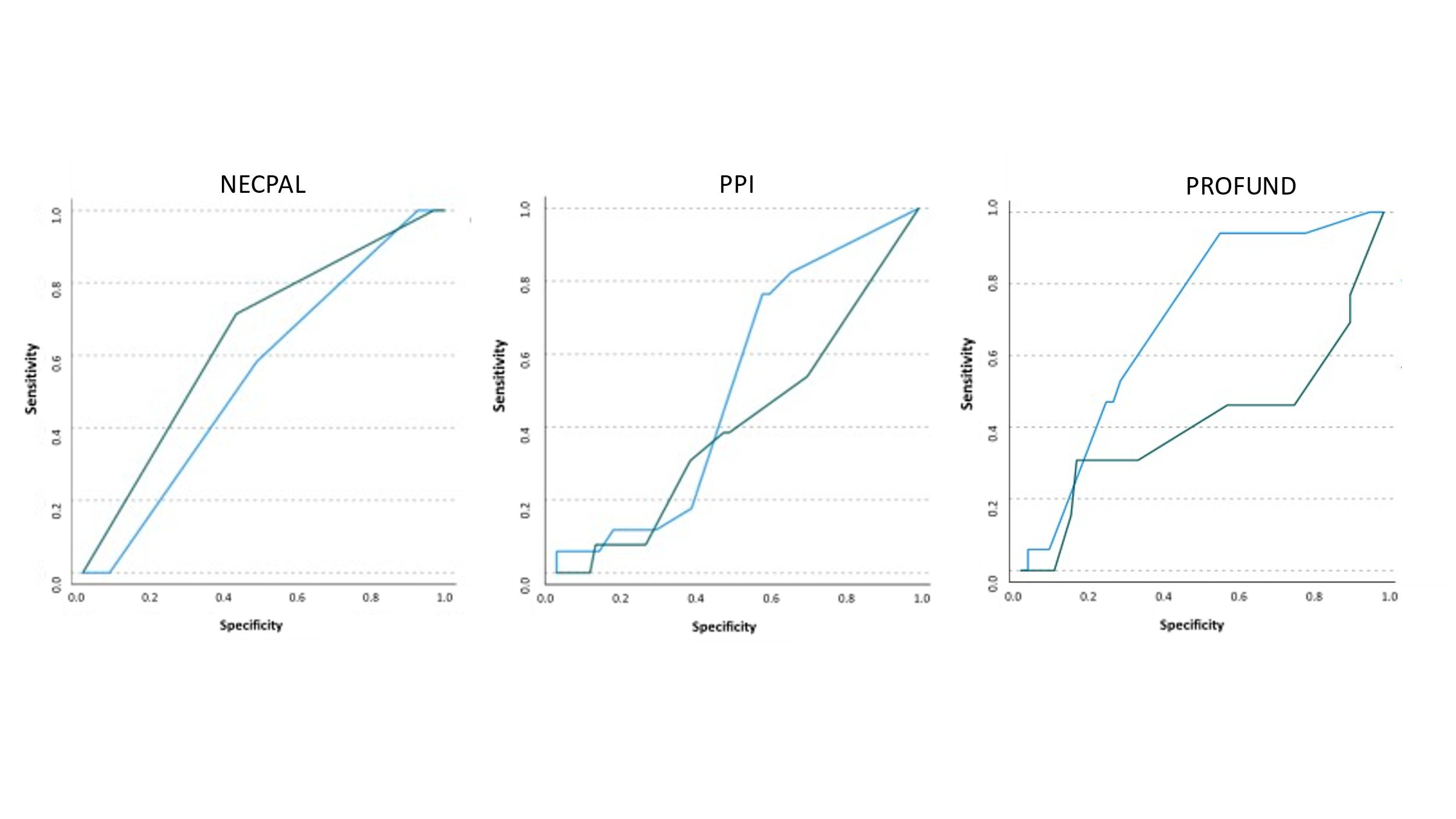


Green = Without dementia; Blue = With dementia

Figure S8: ROC curves for NECPAL ICO-CCOMS©4.0, PPI and PROFUND up to 24 months among residents with and without dementia.


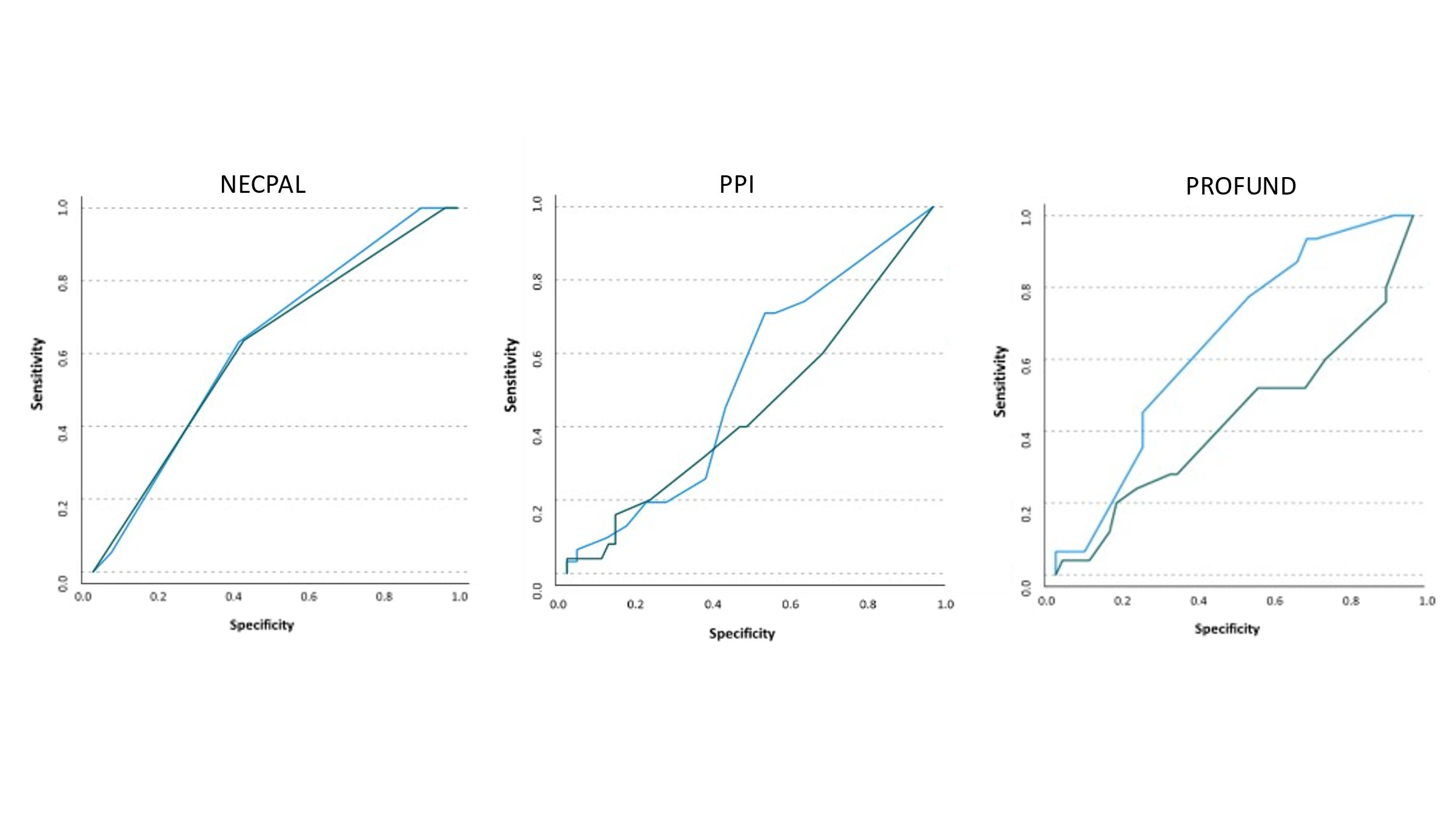


Green = Without dementia; Blue = With dementia
